# Supplementary material for: Complexome profiling of the Chlamydomonas psb28 mutant reveals TEF5 as an early PSII assembly factor
Source: Plant Cell. 2025 Mar 17;37(6):koaf055. doi: 10.1093/plcell/koaf055 (PMC12164590; doi:10.1093/plcell/koaf055)
Supplement: koaf055_Supplementary_Data [file koaf055_supplementary_data.zip › psb28_interactive.zip!/plots/intensity/qualityPlots/peptides/_Cre12.g498950.t1.2.html]

### Cre12.g498950.t1.2

Trivial name:   
SeqCoverage [median]: 17.50  
Mass: 25.90 kDa  
  
 

| Sequence | MissedCleavage | Mass [Da] | wtIntensity | muIntensity | Score |
| --- | --- | --- | --- | --- | --- |
| DLAGLDAPR | 0 | 926.48 | 1996.03 | 2834.81 | 81.30 |
| LHGVVSPETVDEVARR | 1 | 1762.93 | 0.00 | 271.54 | 29.98 |
| VATLGTEPESAGPAGAK | 0 | 1554.79 | 422.86 | 769.81 | 91.96 |
|  |  |  | 2418.9 | 3876.2 |  |
